# All-Season Thermochromic Organogel Polymers for Passive and Sustainable Building Efficiency

**DOI:** 10.1021/acsami.5c22985

**Published:** 2026-02-11

**Authors:** Dixon T. Sin, Samuel Au, Benjamin Dopphoopha, Casper H. Y. Chung, Shuhuai Yao

**Affiliations:** † Department of Mechanical and Aerospace Engineering, 58207 Hong Kong University of Science and Technology, Clear Water Bay, Kowloon 999077, Hong Kong; ‡ Maritime Engineering, School of Engineering, 152304 University of Southampton, Burgess Road, Southampton SO16 7QF, United Kingdom; § HKUST Shenzhen-Hong Kong Collaborative Innovation Research Institute, Futian 518045, Shenzhen, China

**Keywords:** sustainable buildings, smart buildings, building energy saving, thermochromic material, phase change material

## Abstract

Regulating solar heat gain is crucial for reducing heating, ventilation, and air conditioning (HVAC) energy consumption in buildings and promoting sustainable responses to climate change. Current thermochromic materials suffer from poor durability and limited optical modulation. Here, the study presents a durable thermochromic coating based on an organogel-higher alkane (HA) composite. The reversible phase change of HA within the organogel induces light reflection, scattering, and diffraction, while carbon black particles enhance the absorptance modulation, achieving a maximum change of 0.35. For practical application on cement, where a highly reflective layer is applied beneath, the absorptance modulation can reach 0.25, exceeding reported values for other thermochromic systems that could be applied to the roof or wall. The material withstands prolonged UV exposure and repeated thermal cycling without degradation, making it suitable for real-world applications. Simulations incorporating a reflective underlayer demonstrate potential annual HVAC energy savings of up to 3% across diverse climate zones. This work introduces a robust, scalable, and season-adaptive thermochromic coating for sustainable building energy management.

## Graphical abstract

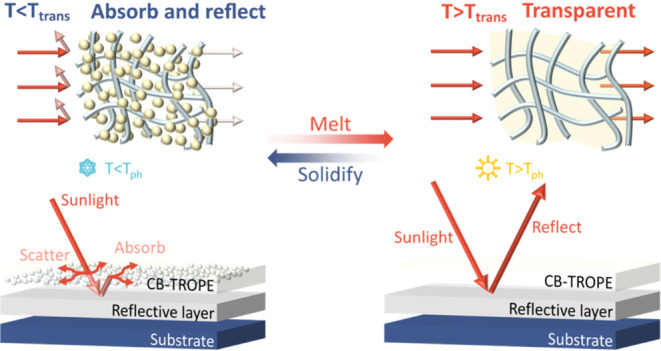

## Introduction

Buildings account for nearly one-third of global energy consumption and over one-quarter of energy-related carbon emissions.^1^ Space heating and cooling account for more than half of household energy use,^2^ with a significant portion of this demand driven by radiative heat exchange,^3^ which is transmitted through windows,^4^ walls, and roofs.^5^ Strategies to regulate solar heat transfer include low-emissivity glass,^6^ hydrogel,^7–9^ electrochromic,^10,11^ infrared radiation regulation,^12,13^ thermochromic^13–15^ coatings, mechanochromic,^16,17^ and radiative cooling.^18,19^ Yet, these technologies face practical constraints: hydrogels require sealed environments, thermochromic paints degrade under UV exposure,^20,21^ and electrochromic and mechanochromic devices rely on active control and external energy input. Radiative cooling is effective only in hot climates, offering limited adaptability in colder regions.

With climate change intensifying weather extremes, there is a growing need for passive, durable, and scalable thermoresponsive coatings that can reflect excess solar radiation in summer while enhancing absorption in winter. Walls and roofs, with their large surface areas^22^ and high absorptivity,^23,24^ represent prime targets for such adaptive regulation. Conventional reflective coatings (e.g., white paints) can minimize heat gain from solar irradiation and reduce cooling loads,^25^ which is particularly beneficial in hot weather. However, they also compromise heating in cold weather, as the reduced heat gain from high-reflectance paint necessitates additional heating to maintain comfortable indoor temperatures.^26^ Ideally, a high change in absorptance can potentially reduce the heating, ventilation, and air conditioning (HVAC) energy consumption in all seasons. Li et al. have demonstrated that such a concept can reduce HVAC energy consumption by 8.2–19.4%.^27^ Yet, this strategy faces challenges related to the energy required for active management and limited scalability. Thus, there is a pursuit of a smart, self-regulating, and scalable wall coating that can provide adaptive thermal regulation and enhance energy efficiency in buildings.

The optimal strategy for regulating irradiation heat gain involves measures that reflect solar irradiation at high temperatures while it absorbs at lower temperatures. A thermoresponsive coating that alters its optical characteristics upon reaching a threshold temperature is a common strategy for this purpose. Conventional thermochromic materials, such as leuco dye, change their solar absorptance through a chemical reaction,^28^ but they will degrade under UV exposure.^29^ Recently, hydrogel (poly­(*N*-isopropylacrylamide), PNIPAM) was utilized as a smart material that blocks irradiation above its lower critical solution temperature (*T*_LCST_) and becomes transparent below this threshold *T*_LCST_.^7,8,14,15^ However, its implementation requires a well-sealed container to prevent PNIPAM from drying, making it impractical for walls and roofs. Despite outstanding solar irradiation regulation performance, PNIPAM may not be suitable for existing traditionally designed buildings due to its limited applicability and feasibility.

To overcome the limitations of existing thermochromic materials, this study proposes a novel approach using thermochromic organogel polymers (TOPs). TOP is a flexible organogel coating, which is an elastomer with high affinity to absorb and utilize the phase change properties of higher alkanes (HA) (i.e., hydrocarbons with nine or more carbon atoms) to regulate solar irradiation under different temperatures.^30–32^ This results in a tunable and thermochromic material that enhances energy efficiency in buildings. Poly­(dimethylsiloxane) (PDMS) was chosen as the organogel due to its favorable mechanical and optical properties, high affinity for oils, and ability to integrate multiple functions.^30–34^ Furthermore, combining TOP with carbon black (CB) particles further increases the change of absorptance, surpassing that of existing thermochromic paints. When integrated with a zirconium dioxide (ZrO_2_) reflecting layer, the coating can achieve higher solar irradiation reflection at temperatures above *T*_ph_ while having higher solar irradiation absorptance at temperatures below *T*_ph_. This dual functionality allows for the regulation of radiation gain in cold weather and the suppression of radiation gain in hot weather. Experimental results indicate that this coating can reduce indoor temperature by 1.2 °C in hot conditions and increase it by 1.1 °C in cold conditions compared to that in bare cement. Building energy simulations further suggest that TOP can reduce annual HVAC energy consumption by up to 3%. Therefore, TOP represents a promising solution for enhancing energy savings in buildings.

## Experimental Section

### Materials

*n*-Hexane (99%) was obtained from RCI Labscan. Pentadecane (98%), hexadecane (98%), heptadecane (99%), and octadecane (98%) were obtained from Aladdin. PDMS (Sylgard 184) was obtained from Dow. Carbon black (SUPER P LI) was obtained from Timical. ZrO_2_ was obtained from Shanghai Yaoyi. Leuco dye was obtained from Glomania. Acrylic resin was obtained from Xuzhou Lvyuan Chemical Co., Ltd. All chemicals were used without further purification.

### Preparation of TOP

HAs were added to the PDMS monomer at a 1:5 weight ratio and then mixed with a magnetic stirring bar at 500 rpm for 2 h. For CB–TOP, carbon black particles were added before stirring, and after 30 min, ultrasonication was performed for 20 min. The mixing and ultrasonication were performed three times for thorough mixing. The PDMS curing agent was added to the precursor, and the mixture was stirred with a magnetic stirring bar at 500 rpm for 10 min. The mixture was then degassed under vacuum for 15 min. The mixture was poured into different molds and cured at 60 °C for 12 h.

### Fabrication of LD–PDMS

Leuco dye was added to the PDMS monomer at a 1 wt % ratio, and then the mixture was stirred using a magnetic stirring bar at 500 rpm for 2 h. The PDMS curing agent was then added to the precursor, and the mixture was stirred with a magnetic stirring bar at 500 rpm for 10 min. The mixture was subsequently degassed under vacuum for 15 min. The mixture was poured into different molds and cured at 60 °C for 12 h.

### Preparation of Cement Samples

Cement powder (Green Island Cement) was mixed with deionized water in a 40:17 weight ratio, then stirred with a magnetic stirring bar at 500 rpm for 10 min. The mixture was poured into molds and cured in room conditions for 12 h.

### Fabrication of ZrO_2_ Reflecting Layer

ZrO_2_ powder was weighed to the amount required to obtain a 60% volume concentration in acrylic resin. The mixture is then mixed with a homogenizer to produce the paint. A layer of paint was then applied to the substrate using a paintbrush and dried on a hot plate at 100 °C. The thickness was measured using a micrometer to ensure that the painted film was 200 μm.

### Reflected Light Differential Interference Contrast (RL-DIC) Microscopy

Microscopy imaging was performed using a microscope (ECLIPSE Ni-U, Brand: Nikon) with a Nomarski prism (RL-DIC Reflected Light Slider, Brand: Nikon). To induce the optical change due to the temperature, a thermal control system was installed beneath the substrate to provide different temperature conditions.

### Photo Properties Characterization

UV–vis–NIR spectral characterizations of substrates were conducted using a Lambda 1050+ spectrometer (PerkinElmer) installed with a 150 mm integrating sphere. For the MIR spectrum, a Vertex 70 Hyperion 1000 (Bruker) was used. By conservation of energy, the sum of absorptance, reflectance, and transmittance must be 1 (α + ρ + τ = 1), so by measuring the transmittance and reflectance of substrates through the machines, the photo characteristics of substrates can be deduced.

### X-ray Diffraction (XRD) Measurement

The XRD measurement was conducted using an X’pert Pro (PANalytical) instrument. During measurement, the samples were maintained at 30 °C (hot state) or 10 °C (cool state), and the 2θ angle ranged from 10 to 60°.

### UV Degradation Experiment

The samples were placed inside a 40-W UV chamber that irradiates with UV light at a peak wavelength of ≈340 nm. The spectral irradiance is shown in Figure S1, which compares the testing lamp and the global solar radiation. The intensity of the UV light is 53 W/m^2,^ and for 24 h of irradiation, the total radiant energy is ≈4.58 MJ/m^2^. Based on the daily average UVA and UVB radiant fluxes obtained by Jacovides et al.,^35^ 15 days of irradiation by the UV chamber is equivalent to 79–270 days depending on the month. Conservatively, it is equivalent to 3 months of UV irradiation under ambient conditions. Every 72 h, the samples will be removed from the chamber, and their UV–vis–NIR photo properties will be measured.

### Temperature Measurement under Solar Simulator

The substrates were placed in a Teflon-insulated box, and a thermocouple was placed under a solar simulator (Photoemission Technology, CT150AAA) in a control room. By adjusting the environmental temperature and applying solar irradiation, the substrates will heat up. The temperatures in the hot state and cool state are 10 and 25 °C, respectively. Tracing the temperature over time helps determine the temperature changes in hot and cool states across different substrates.

### Energy Saving Simulation

OpenStudio (version 3.6.1, an open-source software tool collection that supports whole-building energy modeling https://openstudio.net/) was used to simulate annual HVAC energy consumption. The software calculated energy consumption based on heat transfer across all internal and external building surfaces while accounting for loads within the building (e.g., equipment, lighting, and occupants). HVAC energy consumption across 15 climate zones in the US (as shown in Figure S2) was calculated using the built-in EnergyPlus (version 23.1.0) program. The building model for the simulation is a four-story hotel with a cuboid shape introduced by the Department of Energy (4018.68 m^2^ floor space area, length × width × height = 54.9 × 18.3 × 11.6 m^3^) as shown in Figure S3. The window-to-wall ratio of the model is 0.109, and the material properties are set to the default values downloaded. For bare cement building (baseline), the α_savg_ was set to 0.645 for exterior wall and roof surfaces. For RCB–TOP, the α_savg_ was set to 0.492 and 0.742 for *T* > *T*_ph_ and *T* < *T*_ph_, respectively. For LD–PDMS, the α_savg_ was set to 0.593 and 0.709 for *T* > *T*_ph_ and *T* < *T*_ph_, respectively. By running the model with different climate zones, we can obtain the monthly HVAC energy for cooling or heating. Consider that thermochromic materials can self-regulate their photo properties, so the energy consumption of a building can be calculated by summing the minimum energy consumption across heating and cooling modes.

## Results and Discussion

### Fabrication and Characterization

As illustrated in Figure 1a, the TOP is developed by integrating PDMS organogel with the phase change material HA (see the Experimental Section for detailed fabrication). The absorbed alkanes can regulate the transparency to solar irradiation based on the temperature. To visualize this concept, we performed the reflected light differential interference contrast microscopy (RL-DIC) (see Figure S4) on the PDMS organogel that absorbed hexadecane (C16). From Figure 1b,d, when the temperature is below the phase change temperature of the TOP with C16 (*T* < 10 °C), the crystallization of HA forms micro-sized crystals within the organogel. These crystals scatter, diffract, and reflect incident light, yielding an opaque TOP coating. On the other hand, when the temperature exceeds the phase change temperature (*T* > 10 °C), liquefied HA is reabsorbed by the organogel, which renders the TOP coating transparent to incident light (Figure 1c,1e). A similar mechanism can be observed in poly­(*N*-isopropylacrylamide) (PNIPAM), which acts as a solar energy shield when *T* > *T*_LCST_ and allows solar energy to pass through when *T* < *T*_LCST_.^9^ However, it is important to note that the optical properties of TOP and PNIPAM are opposite in both stages. The mechanism of TOP creates a thermochromic coating that exhibits high transparency when *T* > *T*_ph_ and higher absorptance when *T* < *T*_ph_. For example, when the organogel was cooled from the hot state, the weight-average solar transmittance (τ_savg_) of a 1 mm thick TOP coating decreased from 0.90 to 0.45 while the weight-average absorptance (α_savg_) increased from 0.04 to 0.24. With the conservation of energy when light interacts with a surface, the weight-average reflectance (ρ_savg_) of the TOP increased from 0.06 to 0.31 during the phase change (Figure 1f, calculations can be referred to in Supporting Discussion S1). In the mid-infrared (mid-IR) range (2.5–25 μm; see Figure 1g), there is a noticeable change between the hot and cool states in the 2.5–5 μm region. However, this portion contributes only minimally to the total blackbody radiation at room temperature (298 K). As a result, the infrared transmittance, reflectance, and absorptance remain largely unchanged, with average values (calculations can be referred to in Supporting Discussion S1) of approximately 0.01, 0.01, and 0.98, respectively. These results indicate that the TOP coating primarily modulates optical properties in the solar spectrum (from UV to near-IR), rather than in the mid-IR.

**Figure 1:**
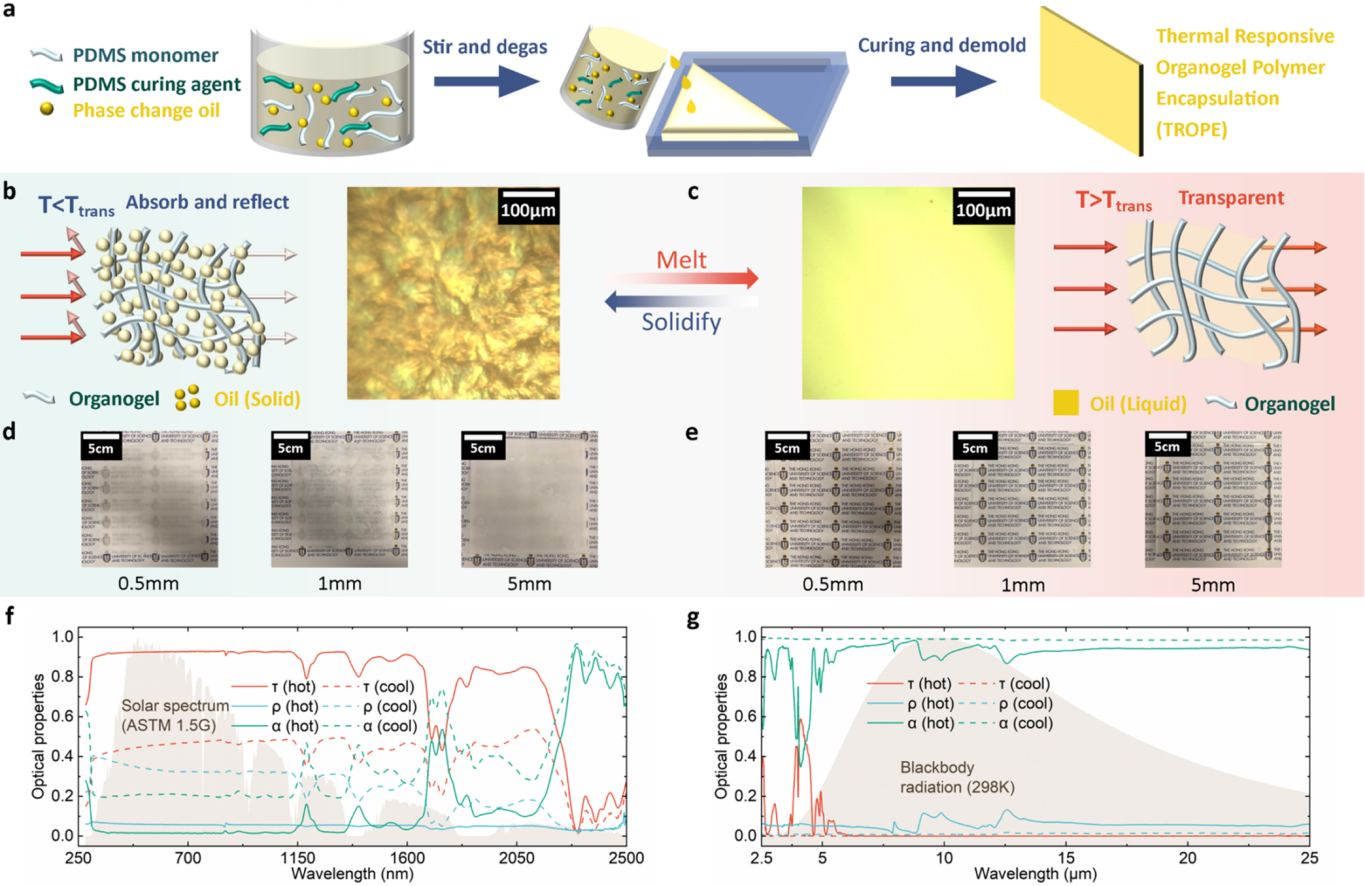
Fabrication and mechanism of TOP. (a) The fabrication procedures of TOP. The schematic illustrations and microscopic images of the molecule structure of TOP when it is lower (b) or higher (c) than the phase transition temperature (*T*_ph_). The photographs of TOP that illustrate the optical appearance of TOP at cool (*T* < *T*_ph_) (d) and hot (*T* > *T*_ph_) (e) states. The spectral absorptance or emissivity of TOP in UV–vis–NIR (f) and MIR (g) spectra.

### Mechanism and Tunable Transition Temperature

During the phase change of HAs, the solidification process leads to the formation of microcrystals that scatter, diffract, and reflect incident light. Taking octadecane (C18) with a thickness of 1 cm as an example, when it is in a solid state, the presence of crystal blocks incident light, resulting in τ_savg_ of approximately 0.104. Conversely, in the liquid state, the absence of crystals allows incident light to pass through, resulting in a τ_savg_ value of about 0.891 (Figure 2a,b). By absorbing the HA within a transparent organogel matrix made of PDMS, the reversible solidification and melting of HA can provide the organogel with thermochromic properties.

**Figure 2:**
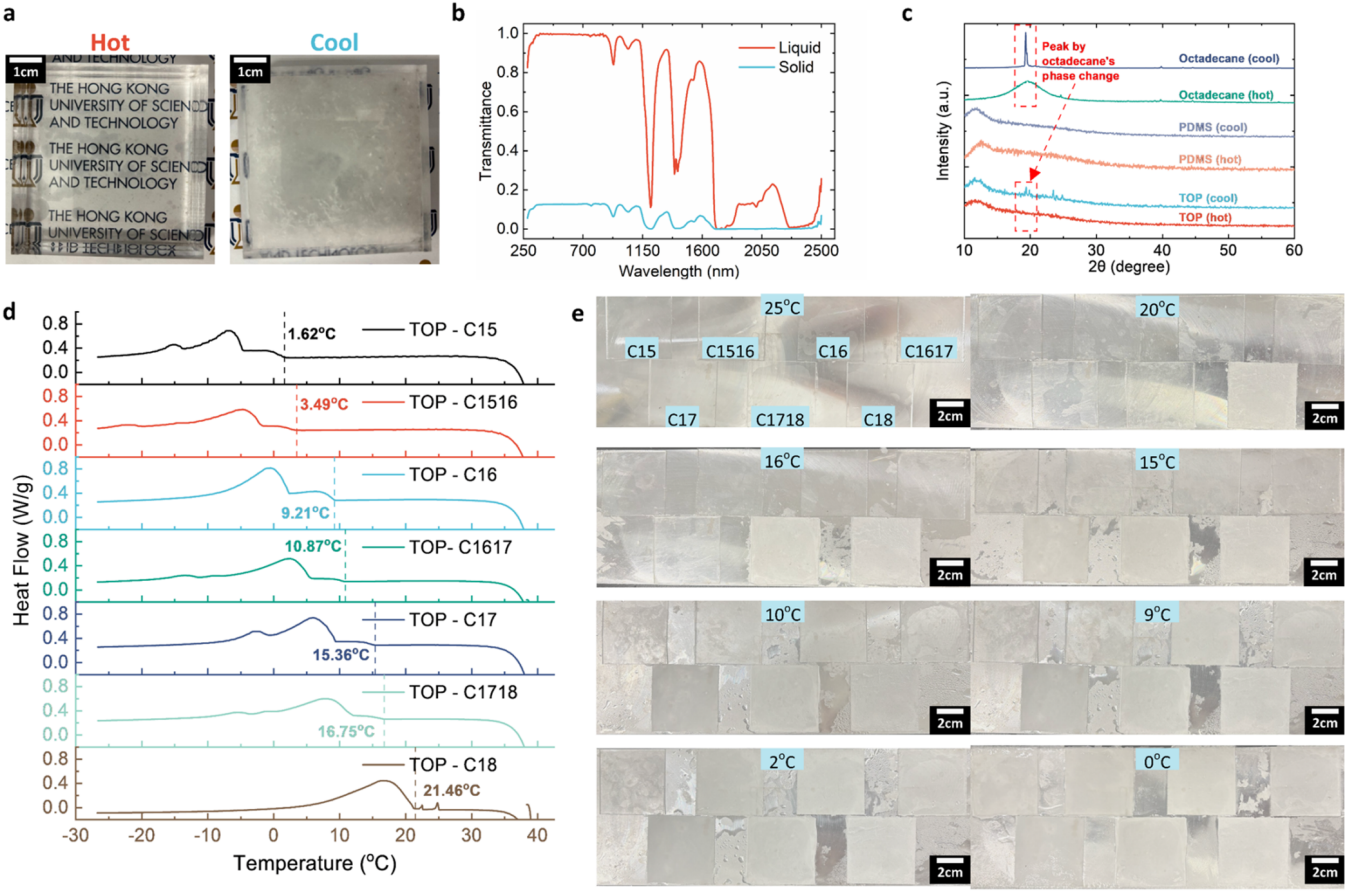
Mechanism and thermal characteristics of TOP. (a) The photo images of octadecane (C18) in hot and cool states. (b) The transmittance of octadecane in liquid and solid states. (c) The X-ray diffraction graph of TOP, octadecane, and plain PDMS in hot and cool states. (d) Graphical summary of the transition temperature of TOP with different HA. (e) The appearance of TOP with different HA at various temperatures. Note that the opaque appearance suggests the HA inside the PDMS matrix has been solidified and forms crystals.

To verify that the microcrystal observed in the cooled state of the organogel is the solidified alkanes, X-ray diffraction (XRD) measurements were carried out on TOP in both the hot state (30 °C) and the cool state (10 °C), with octadecane (C18) and compared to bare PDMS gel (Figure 2c). By normalizing the data, it is evident that a small peak at 2θ ≈ 19° appears when TOP is in the cool state, matching the peak of the solidified C18. This suggests that the thermochromic properties of TOP are attributed to the phase transition of HA within the organogel matrix.

Since HA is responsible for thermochromism, the transition temperature can be adjusted by using different HAs during fabrication. To demonstrate the tunable nature of the transition temperature, various HAs (pentadecane: C15, hexadecane: C16, heptadecane: C17, octadecane: C18, and their mixtures) were employed. To verify that TOP can absorb various HAs, we performed thermogravimetric analysis (TGA) and differential thermal analysis (DTA). The results in Figure S5 showed that TOPs and their corresponding HAs had the same temperature for weight deviation, suggesting the successful entanglement of HAs by TOP.

With different HAs entangled, TOP can achieve tunable phase change temperatures, and the property was measured using differential scanning calorimetry (DSC) (Figures 2d and S6). Noting that C1516, C1617, and C1718 are the 1:1 weight mixture of the corresponding HAs. The results show that the transition temperature of TOP can be varied from 1.62 to 21.5 °C, as depicted in Figure 2e. This suggests that it is possible to achieve a flexible range of transition temperatures by utilizing different HAs. The capability of TOP allows for the incorporation of other alkanes or phase change oils to achieve other thermal-optical properties.

### TOP as Thermochromic Materials

By incorporating an adequate amount of light-absorbing particles, the change in averaged solar absorptance (Δα_savg_) can be further increased since it allows most of the light to pass through in the hot state, while more scattered light in the cool state is absorbed, as illustrated in Figure 3a. In this study, CB was chosen as the additive in the organogel matrix due to its high absorptance for a broad spectrum.^36^ CB was gradually added to TOP to determine the optimal quantity for achieving the highest Δα_savg_ (Figure 3b,3c). The results in Figure 3c indicate that the addition of CB led to increased absorptance in both the hot and the cool states. By calculating the difference (the brown line in Figure 3c), it can be observed that the Δα_savg_ gradually increased until 0.005 wt % of CB, and it yielded the highest Δα_savg_ of 0.349 at 0.005 wt %. Further CB addition (>0.005 wt %) led to a high absorptance in the hot state, limiting the room for Δα_savg_. Therefore, 0.005 wt % of CB was used for the subsequent investigations.

**Figure 3:**
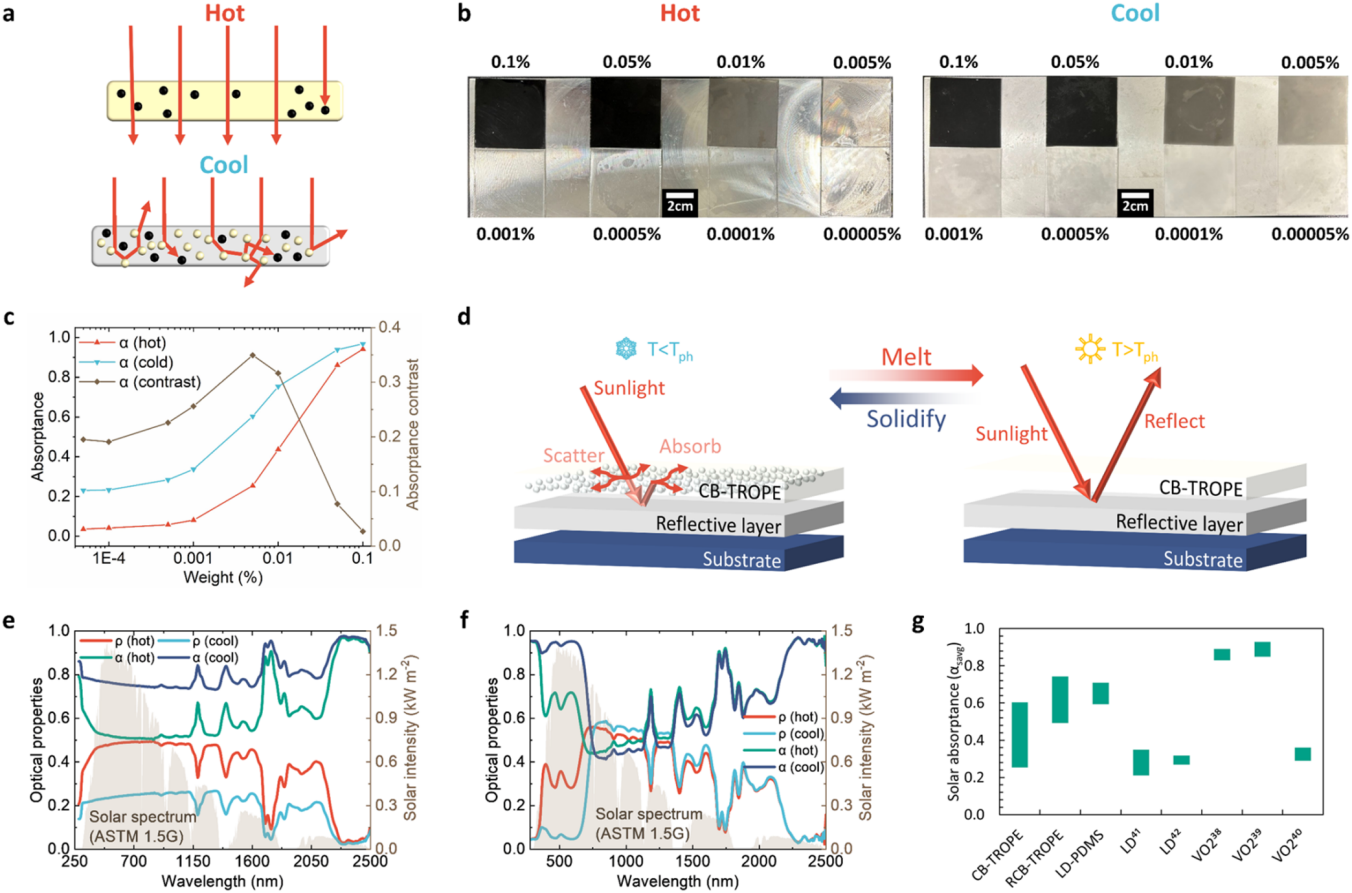
Extensions of TOP. (a) The schematic diagram of the integration of light-absorbing particles and TOP in hot and cool states. (b) The photographs of TOP with different amounts of CB in hot and cool states. (c) The UV–vis–NIR absorptance (α) and change in absorptance (Δα) of CB–TOP. (d) The schematic diagram of the integration of the reflecting layer and CB–TOP. The UV–vis–NIR absorptance (α) and reflectance (ρ) of RCB–TOP (e) and LD–PDMS (f) in hot and cool states. (g) The range of α_savg_ TOP-based and state-of-art thermochromic materials.^38–42^

Despite the increase in Δα_savg_ upon adding CB, CB–TOP (0.005%) remains highly transparent (≈0.75) in the hot state. To reduce heat gain from sunlight in hot weather, the coating is required to reflect sunlight rather than allowing cement to absorb it. A ZrO_2_ reflective coating was introduced underneath the CB–TOP (RCB–TOP, Figure 3d), due to its outstanding solar reflecting properties (up to 0.984).^37^ ZrO_2_ layers with 5 thicknesses were fabricated to compare reflectance and select the optimized configuration that balances the thickness and reflectance. As shown in Figure S7, the reflectance increased significantly from 0.916 to 0.956 as the thickness increased from 100 to 200 μm. Further increasing the thickness yields only a marginal increase in reflectance, and thicker layers may result in cracking. Therefore, 200 μm was selected for the thickness of the ZrO_2_ reflective coating. After the reflecting layer was integrated with CB–TOP (RCB–TOP) on a cement substrate, the ρ_savg_ was measured to be 0.51 in the hot state and 0.26 in the cool state, while the α_savg_ was 0.49 in the hot state and 0.74 in the cool state (Figure 3e). For comparison, leuco dye (the thermochromic dye that can reversibly switch between two chemical forms) was also entangled in PDMS to form leuco-dye-entangled PDMS (LD–PDMS, see Experimental Section for fabrication). With the thermochromic properties endowed by leuco dye, the α_savg_ of LD–PDMS on a cement substrate was 0.59 in the hot state and 0.71 in the cool state, resulting in a Δα_savg_ of 0.12 (Figure 3f). Despite the reduction in Δα_savg_ to 0.25 after integrating with the ZrO_2_ reflecting layer, RCB–TOP still outperforms some reported thermochromic paints, including leuco dye and vanadium dioxide-based paints (VO_2_) (Figure 3g and Table S1).^38–42^ The remarkable optical difference of RCB–TOP at hot and cool temperatures makes it a potential candidate for regulating solar irradiation and for potentially reducing HVAC energy consumption.

### Energy Saving Evaluation of RCB–TOP

To demonstrate the irradiation regulation property of RCB–TOP, a solar heating experiment was conducted in a controlled-environment room to monitor temperature changes in cement substrates with different outermost surfaces. In the experiment, RCB–TOP-coated, LD–PDMS-coated, and bare cement samples (spectral absorptance and reflectance are shown in Figure S8) were exposed to a solar simulator at 1000 W/m^2^ (Photo Emission Technology, CT150AAA), and temperatures were monitored using thermocouples (Figures 4a and S9). In the hot state (25 °C, Figure 4b), the temperature increases observed on the RCB–TOP-coated, LD–PDMS-coated, and bare cement substrates were 4.1, 4.7, and 5.3 °C, respectively. This suggests that the RCB–TOP and LD–PDMS coatings can reduce the temperature increase by 20.9 and 8.2%, respectively. In the cool state (10 °C Figure 4c), the temperature increases on the respective substrates were 6.4, 6.0, and 5.3 °C, indicating that RCB–TOP coating and LD–PDMS coating can increase the temperature increment by 20.6 and 13.7%, respectively. These temperature alterations demonstrate that both RCB–TOP-coated and LD–PDMS-coated cements can modify the photo characteristics in response to temperature. The superior performance of RCB–TOP can be attributed to the α_savg_ values measured in the previous section. The α_savg_ of RCB–TOP-coated cement in the hot state is lower than that of LD–PDMS-coated cement and vice versa in the cool state. These differences enable RCB–TOP-coated cement to reduce heat gain from solar irradiation in the hot state while absorbing more heat in the cool state. The thermochromic properties of these coatings have the potential to help buildings reduce their HVAC energy consumption by adjusting the absorptance and reflectance of walls and roofs in response to the temperature (Figure 4d).

**Figure 4:**
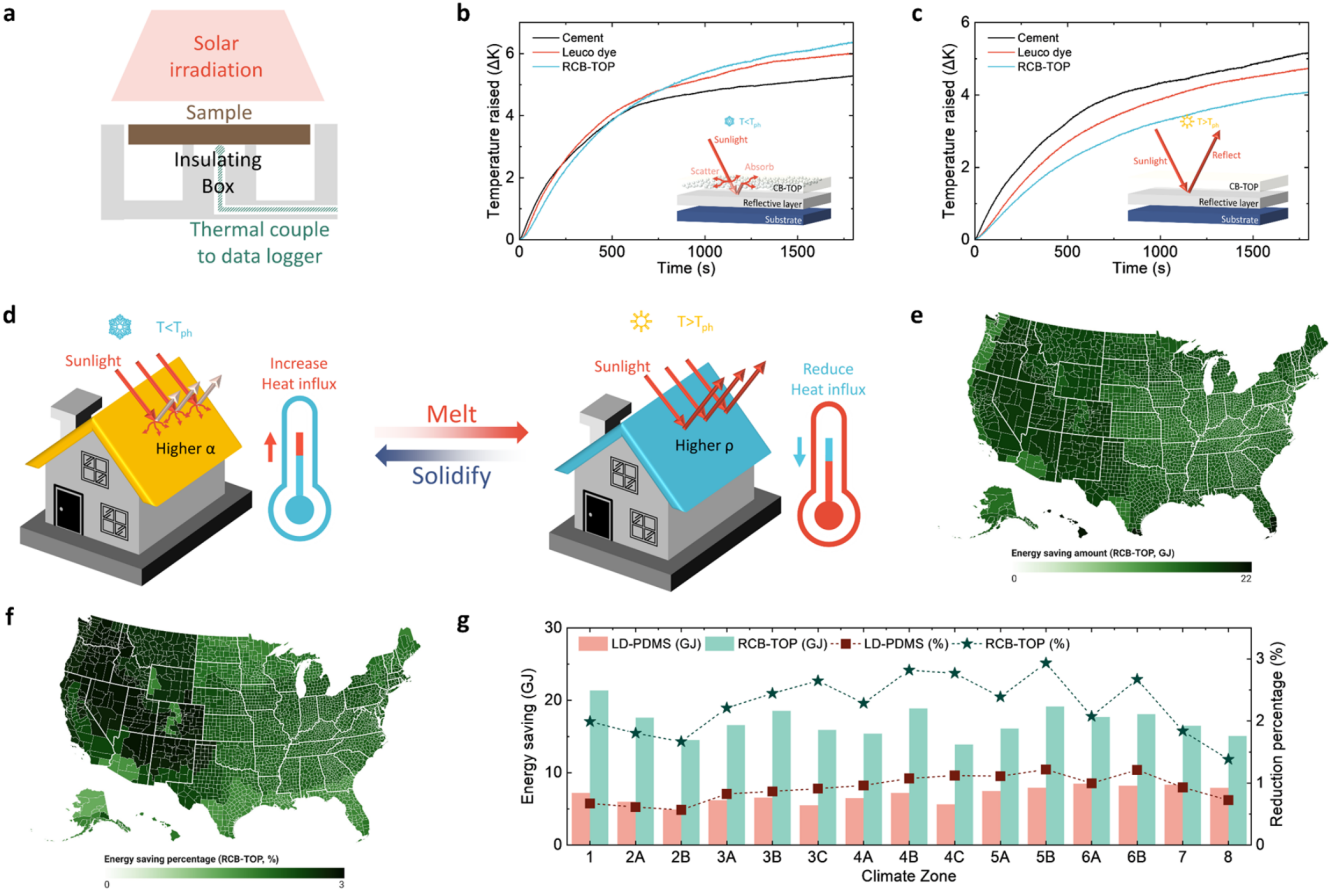
Energy saving demonstration of RCB–TOP. (a) The schematic diagram of the solar heating experiment. The temperature measured by the thermocouple against time in (b) cool and (c) hot states. (d) The schematic diagram illustrating the property of RCB–TOP on the building. The energy saving (e) and percentage energy saving (f) map by RCB–TOP in all 15 climate zones in the US. (g) Annual operational HVAC energy saving and saving percentage of buildings coated with RCB–TOP or LD–PDMS.

In demonstrating the HVAC energy saving potential, as described in the “Energy Saving Simulation,” the optical properties of RCB–TOP-coated and bare cement substrates were imported into OpenStudio. The simulation results indicated that, for the bare cement (baseline) substrate, annual energy consumption ranged from 502 to 1090 GJ across 15 climate zones (Figure S10). However, after applying RCB–TOP as the outermost layer, annual HVAC energy consumption decreased in all climate zones. The maximum annual energy savings achieved with RCB–TOP were 3.0% (Figure 4e,4f). In comparison, the data for LD–PDMS-coated cement were also included in the simulation, and it was found that LD–PDMS could reduce annual HVAC energy consumption by a maximum of 1.2% (Figures 4g and S11).

These results demonstrate that RCB–TOP can be widely applied to buildings under various weather conditions and exhibits superior energy saving performance compared with conventional thermochromic materials, such as leuco dye. With the promising results demonstrated in simulations, the novel thermochromic material, RCB–TOP, could reduce energy consumption in buildings when applied to roofs and walls.

### Durability

In practical applications, the durability of thermochromic materials is crucial, as they are exposed to UV light from the sun and undergo frequent phase transitions due to temperature changes. To assess the durability of TOP, both UV degradation and thermal cycle tests were conducted while tracking the UV–vis–NIR photo characteristics. In the UV degradation test, TOP was compared to LD–PDMS. Leu mparison since it is a common thermochromic material that can be applied to building walls due to its ability to change from colorless (hot state) to black (cool state).^43^ Both samples were exposed to UV irradiation (53 W/m^2^, ≈340 nm) in a closed chamber for 15 days (equivalent to 3 months of sunlight exposure; see Experimental Section for detailed experimental setup). The UV–vis–NIR photo characteristics were measured every 3 days during this period. Figure 5a demonstrates that LD–PDMS turns brownish after UV irradiation, while no observable change is seen in TOP. Quantitative data of the change in absorptance reduction, Δα_savg_ (Figure 5b), further confirm that TOP shows no significant change in Δα_savg_ after UV exposure, whereas LD–PDMS experiences a significant drop from 0.21 to 0.03 in Δα_savg_ after 15 days of UV irradiation. This result can be attributed to the TOP mechanism, which relies on highly stable alkane oils. As long as the HA does not leak from the organogel, the thermochromic properties of TOP will endure. On the other hand, the leuco dye is less chemically stable, making it susceptible to bond breaking caused by UV exposure.^28,29^

**Figure 5:**
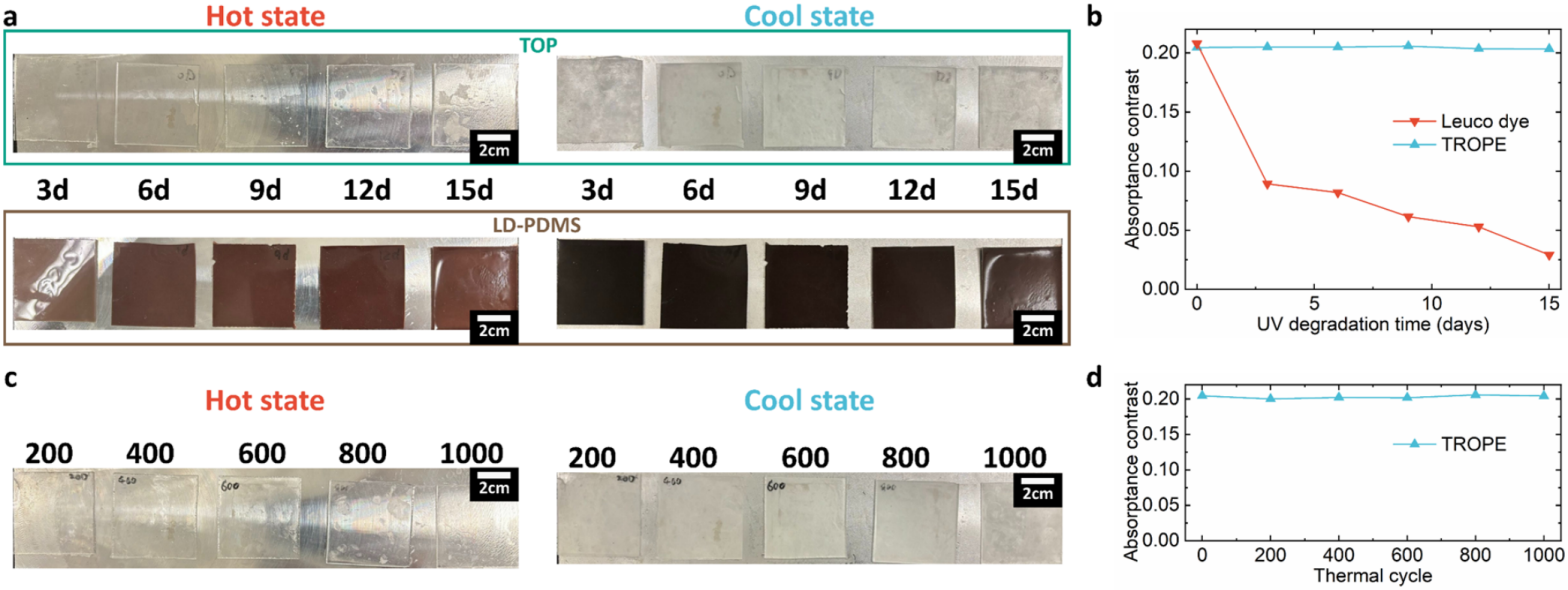
Durability of TOP. (a) The photographs of TOP and LD–PDMS after UV irradiation in hot and cool states. (b) The Δα of TOP and LD–PDMS along the UV durability test. (c) The photographs of TOP after the thermal cycle in hot and cool states. (d) Δα of TOP along the thermal cycle test.

In addition to UV durability, the durability of TOP under thermal cycling was also evaluated. TOP substrates were subjected to repeated cycles of immersion in a 60 °C water bath and ice water (0 °C). The UV–vis–NIR photo characteristics were measured every 200 cycles to assess the degradation of TOP (Figure 5c,d). The results demonstrate that TOP can maintain Δα_savg_ at ≈0.2 after 1000 thermal cycles, indicating its resilience to frequent phase changes. Through the UV irradiation and thermal cycle tests, it is evident that TOP surpasses the limitations of conventional thermochromic paint (leuco dye) and retains its thermochromic properties. This suggests that TOP is a durable thermochromic material that holds promise for applications under sunlight such as on the outer surfaces of buildings.

## Conclusions

This study presents a novel class of thermochromic coatings, thermochromic organogel polymers (TOP) that exhibit durability, tunability, and significant optical modulation. The crystallization of higher alkane inside the organogel facilitates the reflection, scattering, and diffraction of incident light, resulting in an average change in absorptance of approximately 0.2. Since a higher alkane is the functioning part of TOP, tuning the phase change temperature can be achieved by incorporating various higher alkanes, as demonstrated through DSC experiments.

Given that TOP operates through scattering and diffraction mechanisms, the integration of carbon black particles further enhances the average change in absorptance to approximately 0.35. To enable CB–TOP to reflect solar irradiation, a zirconium dioxide reflecting layer was applied beneath it. Although the average change in the absorptance of RCB–TOP was reduced to approximately 0.25, the thermochromic properties still exceeded those of some common thermochromic materials, including vanadium dioxide and leuco dye.

The Δα_savg_ achieved by RCB–TOP can be utilized to regulate the solar irradiation in response to environmental conditions. Chamber experiments confirmed an ≈20% temperature reduction in the hot state (25 °C) and ≈20% temperature increment in the cool state (10 °C). The effective thermal responsive temperature alteration suggests that RCB–TOP can be a promising candidate for HVAC energy savings. Whole-building simulations across 15 U.S. climate zones indicated potential annual HVAC energy savings of up to 3%.

Furthermore, the high stability under UV irradiance and retention of properties after phase change processes underscored the robustness and durability of TOP for real-world use. Combining exceptional thermochromic properties, this innovative TOP-based material holds promise for practical applications in regulating heat gain from solar irradiation on building roofs and walls.

By enabling all-season, self-regulating thermal management, TOP represents a scalable strategy for enhancing building energy efficiency and resilience in the face of climate variability. By effectively regulating heat gain from solar irradiation, it offers potential energy savings and improved thermal comfort for humans. The innovation aligns with the goals of sustainable building design, offering a pathway to reduce global energy demand and emissions from the built environment.

## Supplementary Material



## Notes

Spectral UV irradiance for the UV exposure test (Figure S1); the climate zone map of the US (Figure S2); the 3D drawing of the reference building in the HVAC simulation (Figure S3); experimental setup of reflected light differential interference contrast (Figure S4); the thermogravimetric analysis (TGA) and differential thermal analysis (DTA) graphs of pure PDMS, TOP with various HA, and pure HAs (Figure S5); the detailed differential scanning calorimetry (DSC) graphs of pure PDMS and TOP with various HA and pure HAs (Figure S6); the plot of the reflectance of the ZrO_2_ reflecting layer against its thickness (Figure S7); the UV–vis–NIR spectra of the bare cement substrate (Figure S8); the experimental setup of the solar heating experiment (Figure S9); the energy consumption map of the reference building with bare cement in different climate zones (Figure S10); the energy saving and percentage energy saving map by LD–PDMS in all 15 climate zones in the US (Figure S11); the solar spectral irradiance proposed by ASTM G 173 AM 1.5 (Figure S12); the spectral radiance of a blackbody at 298 K based on Planck’s law (Figure S13); comparison of different thermochromic paints (Table S1); calculation of the weighted average transmittance, reflectance, and absorptance (Supporting Discussion S1) (PDF)
