# Supplementary material for: All-Season Thermochromic Organogel Polymers for Passive and Sustainable Building Efficiency
Source: ACS Appl Mater Interfaces. 2026 Feb 11;18(9):14221–9. doi: 10.1021/acsami.5c22985 (PMC12983202; doi:10.1021/acsami.5c22985)
Supplement: Supplementary file 1 [file am5c22985_si_001.pdf]

Supporting Information

All-Season Thermochromic Organogel Polymers for Passive and Sustainable Building Efficiency

Authors: Dixon T. Sin[1,2]*, Samuel Au[1], Benjamin Dopphoopha[1], Casper H.Y. Chung[1], Shuhuai Yao[1,3]*

[1] Department of Mechanical and Aerospace Engineering, Hong Kong University of Science and Technology, Clear Water Bay, Kowloon, Hong Kong, 999077

[2] Maritime Engineering, School of Engineering, Burgess Road, University of Southampton, Southampton, United Kingdom, SO16 7QF

[3] HKUST Shenzhen-Hong Kong Collaborative Innovation Research Institute, Futian, Shenzhen, China, 518045

*Corresponding authors' E–mail:

Dixon T. Sin: t.sin@soton.ac.uk

Shuhuai Yao: meshyao@ust.hk

Contents

Supporting Figures 1-9

Supporting Table 1

Supporting Discussion 1

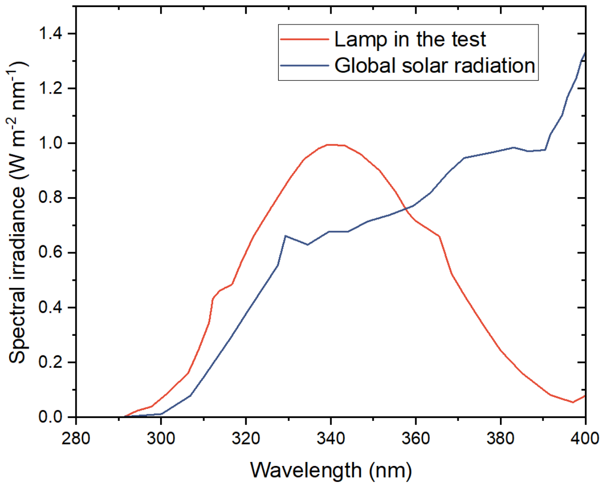

Figure S1 The spectral irradiance of the UV chamber used for the UV exposure test

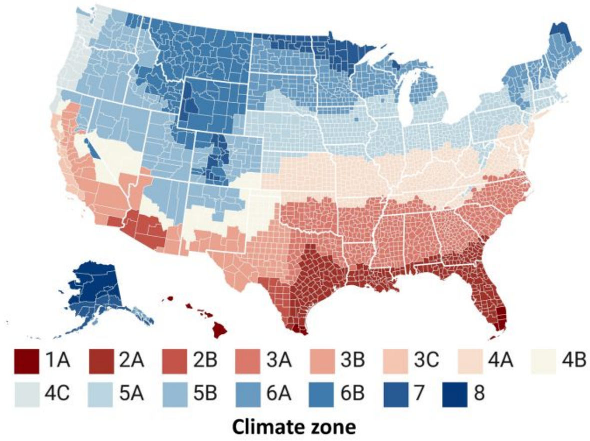

Figure S2 Climate zone map of the US. The climate zones are distinguished based on the temperature and the humidity.

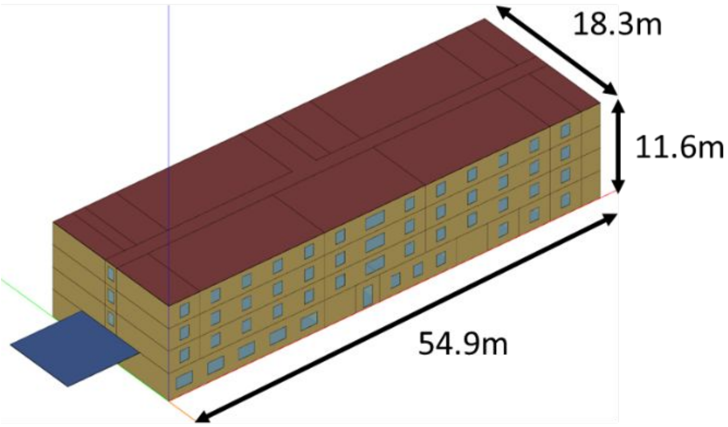

Figure S3 The 3D drawing of the reference building in the HVAC energy consumption simulation.

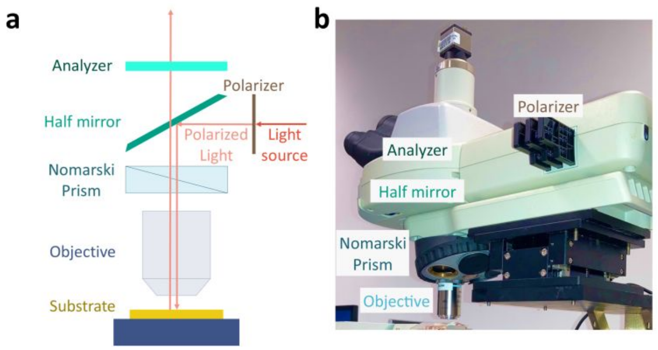

Figure S4 Experimental setup of reflected light differential interference contrast (RL-DIC). **a,** Schematic image. **b,** Photo image.

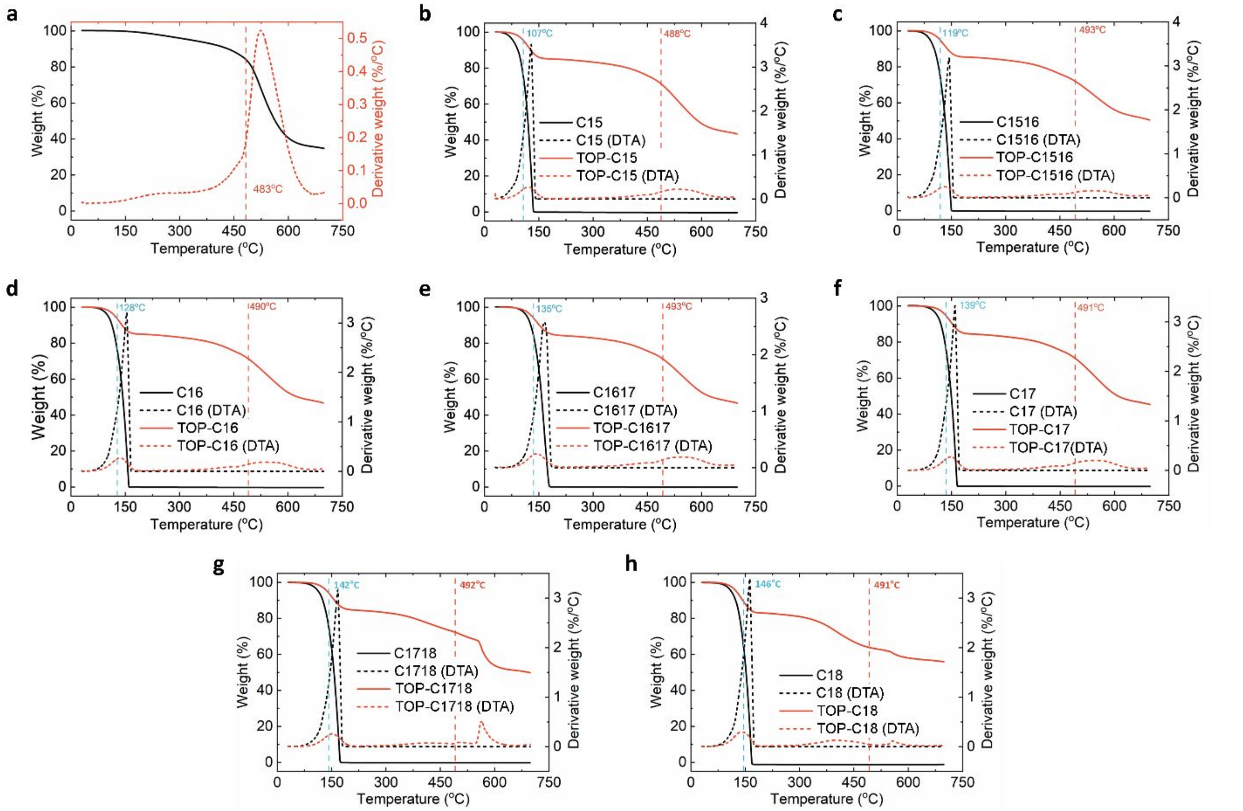

Figure S5 The thermogravimetric analysis (TGA) and differential thermal analysis (DTA) graphs of pure PDMS, TOP with various HA and pure HAs. a, PDMS. **b,** Pentadecane (C15) and TOP with C15. **c,** Pentadecane and hexadecane mixture (C1516, 1:1 weight ratio) and TOP with C1516. **d,** Hexadecane (C16) and TOP with C16. **e,** Hexadecane and heptadecane mixture (C1617, 1:1 weight ratio) and TOP with C1617. **f,** Heptadecane (C17) and TOP with C17. **g,** Heptadecane and Octadecane mixture (C1718, 1:1 weight ratio) and TOP with C1718. **h,**

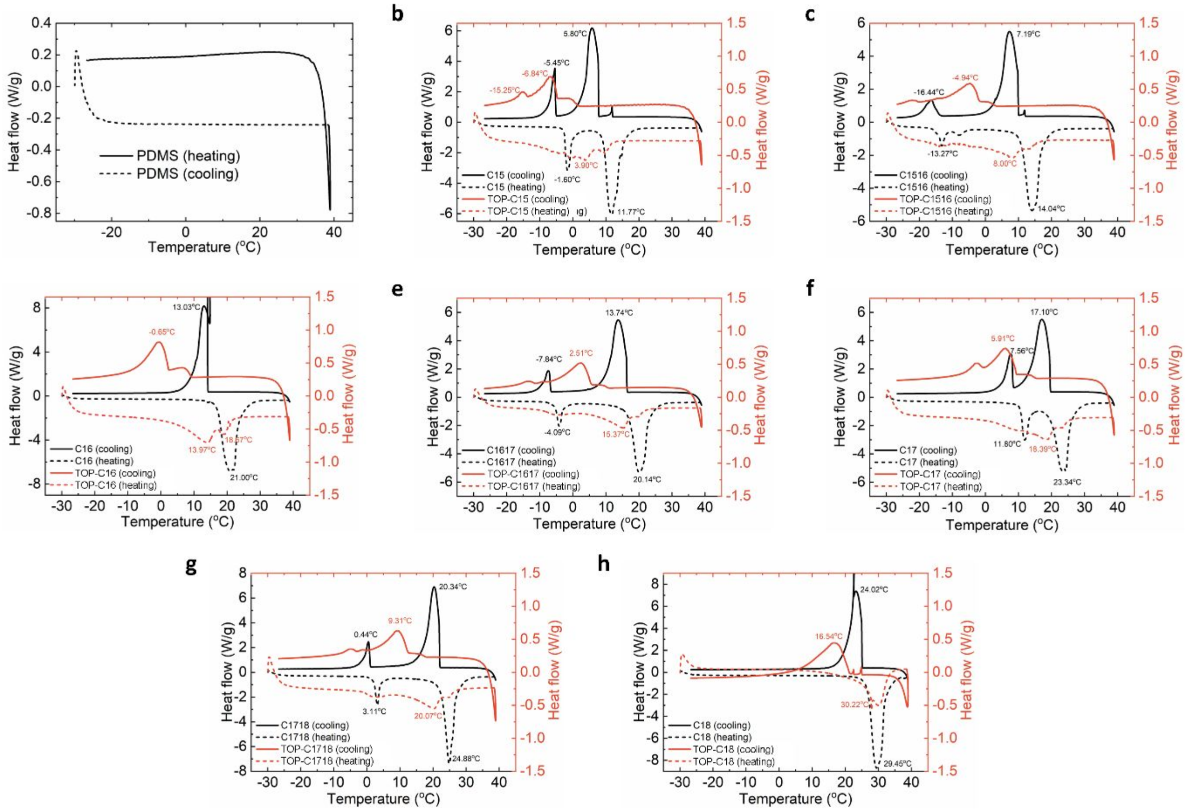

Figure S6 The detailed differential scanning calorimetry (DSC) graphs of pure PDMS and TOP with various HA and pure HAs. a, PDMS. **b,** Pentadecane (C15) and TOP with C15. **c,** Pentadecane and hexadecane mixture (C1516, 1:1 weight ratio) and TOP with C1516. **d,** Hexadecane (C16) and TOP with C16. **e,** Hexadecane and heptadecane mixture (C1617, 1:1 weight ratio) and TOP with C1617. **f,** Heptadecane (C17) and TOP with C17. **g,** Heptadecane and octadecane mixture (C1718, 1:1 weight ratio) and TOP with C1718. **h**, Octadecane (C18) and TOP with C18.

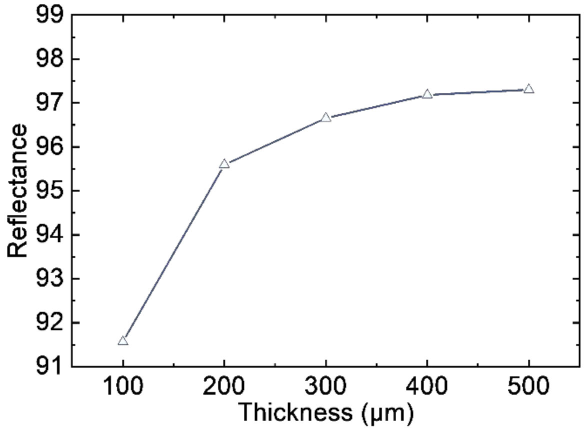

Figure S7 The plot of the reflectance of ZrO_2 reflecting layer against its thickness

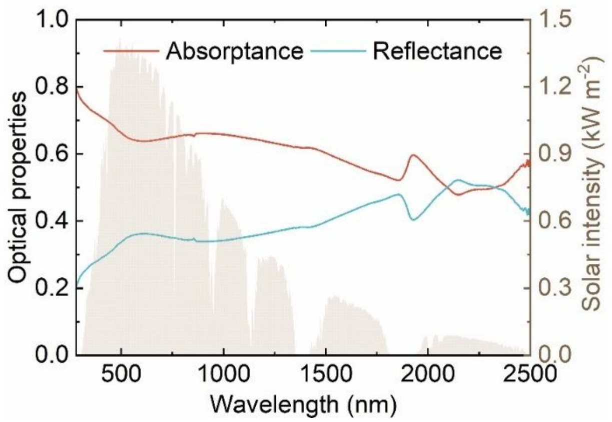

Figure S8 The UVVISNIR spectra of the bare cement substrate.

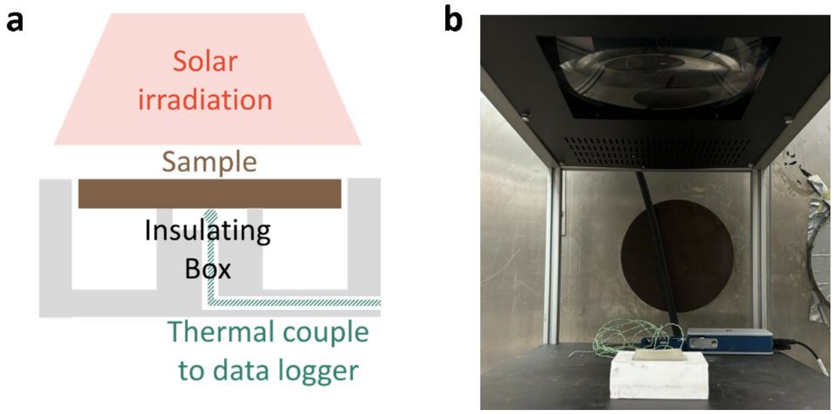

Figure S9 The experimental setup of the solar heating experiment. (a) Schematic figure. (b) Photo image.

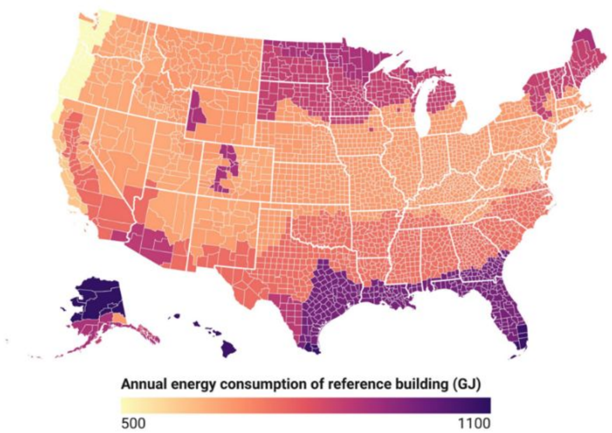

Figure S10 The energy consumption map of the reference building with bare cement in different climate zones.

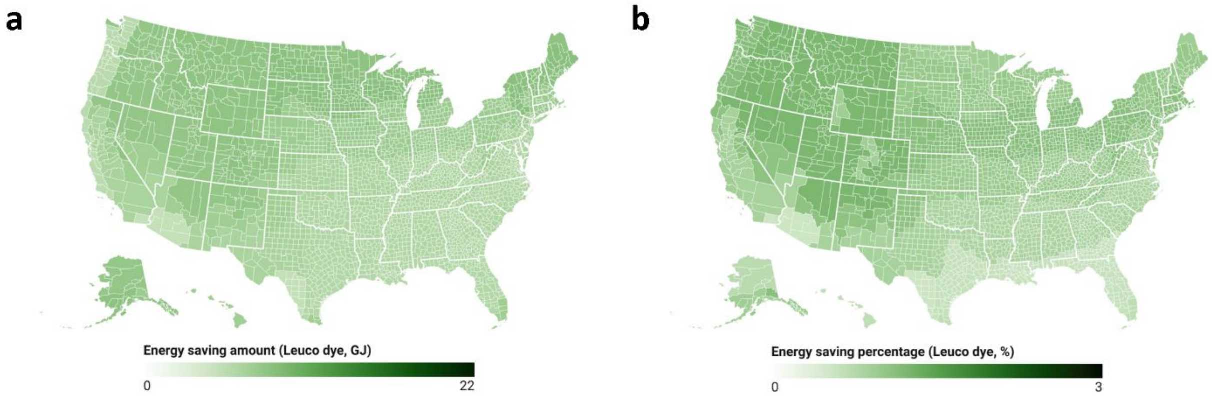

Figure S11 The energy saving (a) and percentage energy saving (b) map by LD–PDMS in all 15 climate zones in the US.

Table S1 Comparison of different thermochromic paints, corresponding to Fig. 4g.

Strategy	Switching material	Absorptance range	Absorptance changes
Leuco dye [1]	Leuco dye	0.21 – 0.35	0.14
Leuco dye [2]	Leuco dye	0.269 – 0.317	0.048
Vo_2 [3]	Vo_2	0.83 – 0.89	0.06
Vo_2 [4]	Vo_2	0.849 – 0.928	0.079
Vo_2 [5]	Vo_2	0.29 – 0.36	0.07
LD–PDMS (this work)	Leuco dye	0.593 – 0.709	0.116
CB – TOP (this work)	Higher alkane	0.254 – 0.603	0.349
RCB–TOP (this work)	Higher alkane	0.492 – 0.742	0.250

Supporting Discussion 1: Weighted average transmittance (τ_{avg}), reflectance (ρ_{avg}), and absorptance (α_{avg})

The approach to obtain the weight average transmittance (τ_{avg}), reflectance (ρ_{avg}), and absorptance (α_{avg}) is introducing weighted function ($\tau(\lambda)$, $\rho(\lambda)$, and $\alpha(\lambda)$ for transmittance, reflectance, and absorptance, respectively) to the integration of energy for a reference spectrum (solar spectrum (280nm to 2500nm) or MIR spectrum (2.5 μm to 25 μm), thus, to obtain the energy transmitted (E_τ), reflected (E_ρ), and absorbed (E_α):

$$E_\tau = \int_{\lambda_1}^{\lambda_2} \tau(\lambda) I(\lambda) d\lambda \quad (1)$$

$$E_\rho = \int_{\lambda_1}^{\lambda_2} \rho(\lambda) I(\lambda) d\lambda \quad (2)$$

$$E_\alpha = \int_{\lambda_1}^{\lambda_2} \alpha(\lambda) I(\lambda) d\lambda \quad (3)$$

where $I(\lambda)$ is the spectral irradiance.

Here, in the solar spectrum, $I(\lambda)$ refers to the American Society for Testing and Materials (ASTM) G-173 spectra data with an air mass 1.5 (**Figure S12**). Combining the spectral irradiance and the photo characteristics results obtained from UVVISNIR, the τ_{savg}, ρ_{savg}, and α_{savg} can then be obtained by dividing $E_{s\tau}$, $E_{s\rho}$, and $E_{s\alpha}$ by total energy ($E_s = E_{s\tau} + E_{s\rho} + E_{s\alpha}$) respectively.

$$\tau_{savg} = \frac{E_{s\tau}}{E_s},\ \rho_{savg} = \frac{E_{s\rho}}{E_s},\ \alpha_{savg} = \frac{E_{s\alpha}}{E_s} \quad (4)$$

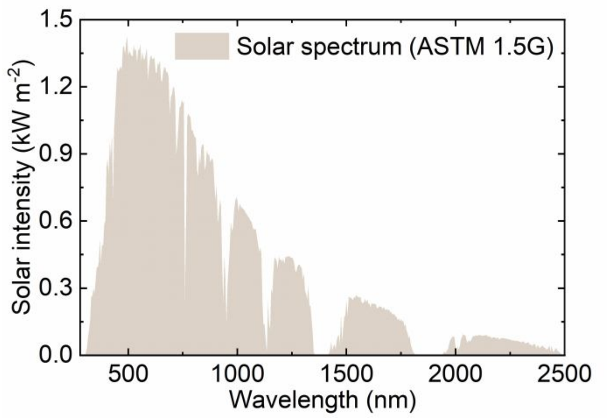

Figure S12 The solar spectral irradiance proposed by ASTM G173 AM 1.5

For MIR spectrum, the spectral radiance can be calculated based on Planck's law (Eq. 5), which is depended on the object's temperature. For an object in 298K (room condition), the spectral radiance is as shown in **Figure S13**. Therefore, the τ_{avg}, ρ_{avg}, and α_{avg} in MIR range can be obtained by inserting the I(λ) with values from Planck's law.

$$B_{\lambda}(T) = \frac{2hc^2}{\lambda^5}\frac{1}{e^{\frac{hc}{\lambda k_B T}} - 1} \qquad 5$$

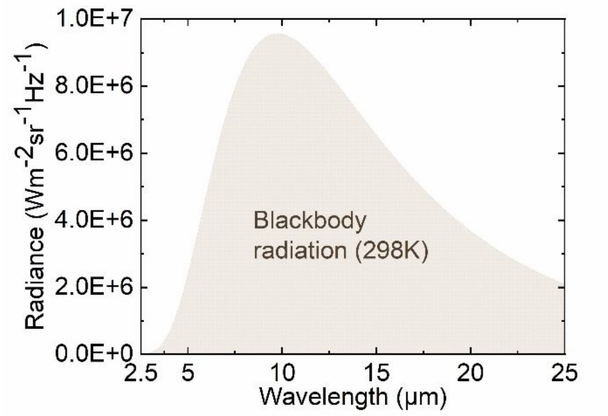

Figure S13 The spectral radiance of black body at 298K based on Planck's law

Reference

1. Berardi, U., Garai, M. & Morselli, T. Preparation and assessment of the potential energy savings of thermochromic and cool coatings considering inter-building effects. *Solar Energy* 209, 493–504 (2020).

2. Perez, G., Sirvent, P., Sanchez-Garcia, J. A. & Guerrero, A. Improved methodology for the characterization of thermochromic coatings for adaptive façades. *Solar Energy* 230, 409–420 (2021).
3. Ao, X. *et al.* Self-adaptive integration of photothermal and radiative cooling for continuous energy harvesting from the sun and outer space. *Proceedings of the National Academy of Sciences* 119, (2022).
4. Sun, K. *et al.* VO2 metasurface smart thermal emitter with high visual transparency for passive radiative cooling regulation in space and terrestrial applications. *Nanophotonics* 11, 4101–4114 (2022).
5. Krammer, A., Matilainen, A., Pischow, K. & Schüler, A. VO2:Ge based thermochromic solar absorber coatings. *Solar Energy Materials and Solar Cells* 240, 111680 (2022).
